# Peroxidase-Mimicking Activity of Nanoceria for Label-Free Colorimetric Assay for Exonuclease III Activity

**DOI:** 10.3390/ijms241512330

**Published:** 2023-08-02

**Authors:** Hyogu Han, Jae Hoon Jeung, Se Hee Jang, Chang Yeol Lee, Jun Ki Ahn

**Affiliations:** 1 Material & Component Convergence R&D Department, Korea Institute of Industrial Technology (KITECH), Ansan 15588, Republic of Korea; ninehyo@kitech.re.kr (H.H.); qaz2758@kitech.re.kr (J.H.J.); seahee2150@kitech.re.kr (S.H.J.); 2 Department of Chemistry, Gangneung-Wonju National University, Gangneung 25457, Republic of Korea; 3 Department of Medical Device Engineering and Management, College of Medicine, Yonsei University, Seoul 03722, Republic of Korea; 4 Bionanotechnology Research Center, Korea Research Institute of Bioscience and Biotechnology (KRIBB), Daejeon 34141, Republic of Korea

**Keywords:** cerium oxide nanoparticle, peroxidase-mimicking activity, exonuclease III, colorimetric assay

## Abstract

We present a novel label-free colorimetric method for detecting exonuclease III (Exo III) activity using the peroxidase-mimicking activity of cerium oxide nanoparticles (nanoceria). Exo III, an enzyme that specifically catalyzes the stepwise removal of mononucleotides from the 3′-OH termini of double-stranded DNA, plays a significant role in various cellular and physiological processes, including DNA proofreading and repair. Malfunctions of Exo III have been associated with increased cancer risks. To assay the activity of Exo III, we applied the previous reports in that the peroxidase-mimicking activity of nanoceria is inhibited due to the aggregation induced by the electrostatic attraction between DNA and nanoceria. In the presence of Exo III, the substrate DNA (subDNA), which inhibits nanoceria’s activity, is degraded, thereby restoring the peroxidase-mimicking activity of nanoceria. Consequently, the 3,3′,5,5′-tetramethylbenzidine (TMB) substrate is oxidized, leading to a color change from colorless to blue, along with an increase in the absorbance intensity. This approach enabled us to reliably detect Exo III at a limit of detection (LOD) of 0.263 units/mL across a broad dynamic range from 3.1 to 400 units/mL, respectively, with an outstanding specificity. Since this approach does not require radiolabels, complex DNA design, or sophisticated experimental techniques, it provides a simpler and more feasible alternative to standard methods.

## 1. Introduction

Exonucleases represent a class of enzymes with a unique ability to catalyze the cleavage of DNA molecules through hydrolyzing the phosphodiester bonds, thereby playing a pivotal role in DNA digestion. Notably, these enzymes function at either the 3′-terminus or the 5′-terminus of the DNA sequence, dependent on the type of exonuclease [1,2]. One prominent type of exonuclease is exonuclease III (Exo III), a well-known member of the exonuclease enzyme family. This particular enzyme contributes to the sequential removal of mononucleotides from the 3′-hydroxyl termini of double-stranded DNA, thus aiding in DNA disassembly via a methodical catalytic process [3]. The 3′-5′ activity of Exo III exhibits a crucial role across several critical cellular and physiological processes, including DNA proofreading and repair [4,5,6]. When defects occur in 3′-5′ exonucleases, cells may process transcription and translation incorrectly, which can lead to a lack of protection against cancer, particularly when exposed to long periods of stress [7]. Consequently, it is essential to develop reliable analytical methods for accurately measuring the activity of 3′-5′ exonucleases [8].

Gel electrophoresis and radiolabeling serve as the standard techniques for detecting 3′-5′ exonuclease activity. However, despite their widespread use, both methods are time-consuming, labor intensive, and pose safety risks during the detection process [9,10]. Recently, alternative methods for detecting 3′-5′ exonuclease activity have been developed, such as fluorescence-based methods utilizing the G-quadruplex [11,12,13,14,15] and FRET [16,17,18,19]. In addition, a detection method using metal nanoparticles, including copper nanoparticles [20,21], has been developed for detecting 3′-5′ exonuclease activity. However, these techniques still present several drawbacks, such that they necessitate complicated preparation procedures and the use of modified fluorescent probes, making them less straightforward and convenient.

Since the discovery of the peroxidase-mimicking activity of magnetic nanoparticles (MNPs) [22], there has been increasing interest in nanoscaled peroxidase mimetics and their potential applications in bioanalysis. This includes a multitude of metal nanoparticles, such as gold nanoparticles (AuNPs) [23,24], platinum nanoparticles (PtNPs) [25,26], iron oxide nanoparticles (Fe_3_O_4_ NPs) [27,28], cupric oxide nanoparticles (CuO NPs) [29], and cerium oxide nanoparticles (CeO_2_ NPs, also termed as nanoceria) [30,31,32].

Remarkably, cerium oxide nanoparticles (nanoceria) exhibiting oxidase-like activity present exceptional stability and catalytic efficiency for substrate oxidation, even in the absence of supplemental oxidizing agents [30,31]. Previous studies have reported that the peroxidase-mimicking activity of nanoceria can be regulated using nucleic acids. This regulation occurs through electrostatic interactions that bind the nucleic acids to the nanoparticles. The subsequent aggregation of nanoceria, induced by this binding, incites a decline in their catalytic activity [33,34,35]. 

In this study, we have devised a new label-free colorimetric detection method for Exo III activity utilizing the peroxidase-mimicking property of nanoceria. Our strategy relies on inhibiting nanoceria’s peroxidase-mimicking activity through substrate DNA (subDNA) binding. The absence of Exo III allows the subDNA to bind to nanoceria, causing aggregation due to electrostatic attraction. This binding significantly hinders the efficient oxidation of the 3,3′,5,5′-tetramethylbenzidine (TMB) substrate, limiting direct contact and resulting in a low absorbance intensity due to the production of a small quantity of oxidized TMB. However, the presence of Exo III degrades the subDNA, which enhances the peroxidase-mimicking activity of nanoceria and promotes the oxidation of the TMB substrate. This induces a color change from colorless to blue, which consequently results in an increase in the absorbance intensity.

## 2. Results and Discussion

### 2.1. Exo III Detection Based on Nanoceria

**Figure 1:**
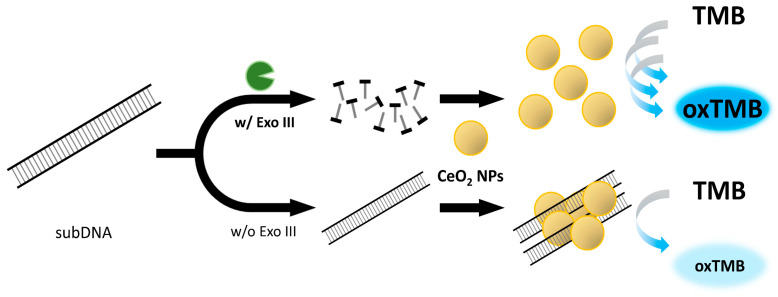
Schematic illustration of the label-free colorimetric assay for exonuclease III activity based on cerium oxide nanoparticles (nanoceria).

The overall procedure for the nanoceria-based label-free colorimetric detection of Exo III has been illustrated in Figure 1. Exo III exhibits specificity in catalyzing the stepwise removal of mononucleotides from the 3′-OH termini of dsDNA. We employed a dsDNA with a length of 200 bp as the substrate for Exo III. In the absence of Exo III, the subDNA binds to the surface of the nanoceria and causes aggregation due to the electrostatic attraction between the positive charge on the surface of the nanoceria and the negative charge on the phosphate backbone. Consequently, the binding effects of the subDNA greatly hinder the efficient oxidation of the TMB substrate by restricting direct contact between the nanoceria and the TMB substrate. This limitation results in the production of a small quantity of oxidized TMB, consequently leading to a low absorbance intensity. However, in the presence of Exo III, subDNA is digested by the specific activity of Exo III. As a result, nanoceria actively facilitates the oxidation of TMB, leading to a recovered absorbance intensity. 

### 2.2. Feasibility of Exo III Detection

**Figure 2:**
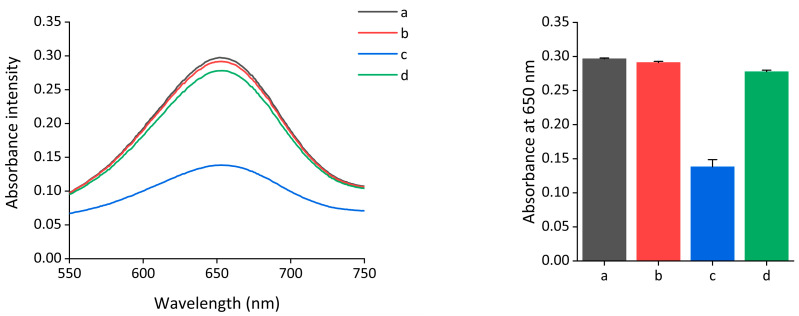
Feasibility of the nanoceria-based colorimetric assay of Exo III. Absorption spectra were measured under the following conditions: a: nanoceria, b: nanoceria + Exo III, c: nanoceria + subDNA, and d: nanoceria + subDNA + Exo III. The nanoceria, Exo III, and subDNA concentrations were 0.375 wt%, 400 units/mL, and 400 nM, respectively. The error bars indicate the standard deviations obtained from triplicate measurements.

To demonstrate the feasibility of the colorimetric assay of Exo III, the absorbance intensity spectra of oxidized TMB under different conditions were obtained. As shown in Figure 2, a highly retained absorbance signal was observed when only nanoceria were present (line a), and a negligible decrease in the absorbance intensity was observed even when Exo III was present together (line b). In contrast, a notable reduction in the absorbance intensity was observed in the presence of both the nanoceria and subDNA (line c). This indicates that the peroxidase-mimicking activity of nanoceria was reduced by the interaction between the nanoceria and subDNA. However, the highly recovered absorbance intensity was measured when Exo III was added to the nanoceria and subDNA. These results indicate that Exo III promotes the degradation of the subDNA, which in turn regulates the peroxidase-mimicking activity of nanoceria. This led to a change in the absorbance intensity by Exo III, as depicted in the mechanism displayed in Figure 1.

**Figure 3:**
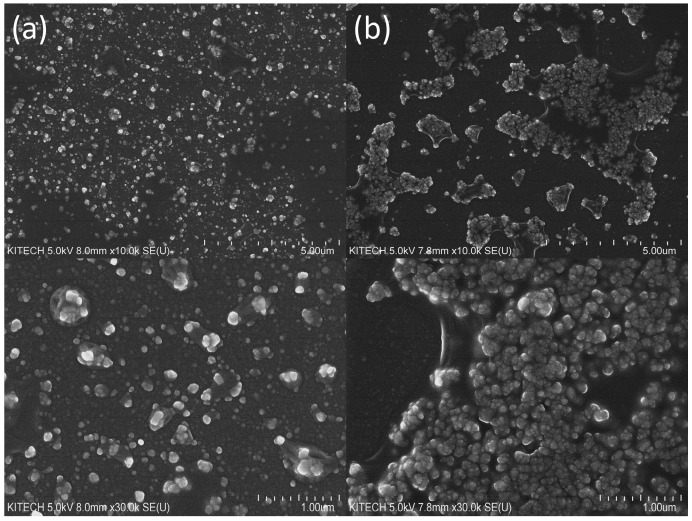
SEM images of nanoceria in the (**a**) absence and (**b**) presence of the subDNA. The concentrations of nanoceria and subDNA were 0.0375 wt% and 40 nM, respectively.

Next, we investigated the inhibitory effects of DNA on the oxidase-mimicking activity of nanoceria. From analyzing scanning electron microscopy (SEM) images to evaluate the surfaces of nanoceria (Figure 3) and utilizing dynamic light scattering (DLS) to measure particle size distributions (Figure S1a), it was observed that the aggregation of nanoceria was significantly enhanced when incubated with the subDNA compared to those not exposed to subDNA treatment. Furthermore, it was confirmed that the zeta potential of nanoceria decreased from 7.03 mV to −1.078 mV, respectively, following aggregation induced by the subDNA (Figure S1b). These results align well with the outcomes that have been reported in previous reports [33,34,35].

### 2.3. Determination of the Exo III Detection System

**Figure 4:**
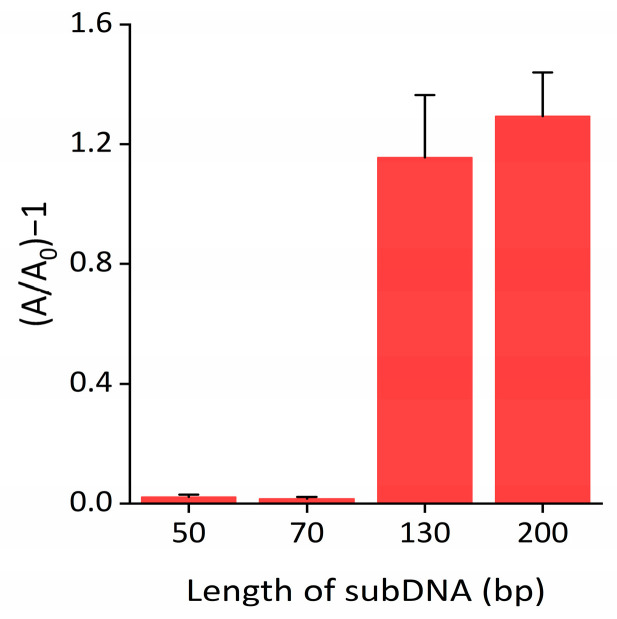
Optimization of subDNA length. The absorbance intensity ratio is defined as (A/A_0_)−1, where A and A_0_ indicate the absorbance intensity at 650 nm with and without the Exo III, respectively. The error bars indicate the standard deviations obtained from triplicate measurements.

To maximize the performance of the Exo III detection system, we optimized the reaction conditions by examining the absorbance intensity defined as (A/A_0_)−1, where A_0_ and A represent the absorbance intensity at 650 nm in the absence and presence of Exo III, respectively. As shown in Figure 4 and Figure S2, the optimal absorbance intensity ratio was observed for a subDNA length of 200 bp. SubDNAs shorter than 130 bp interacted weakly with nanoceria and were not able to sufficiently inhibit peroxidase-mimicking activity. Furthermore, the absorbance signal declined as the concentration of subDNA increased up to 400 nM, after which it reached a plateau (Figure S3). The results in Figure S4 reveal that the optimal concentration of nanoceria was 0.375 wt%. As shown in Figures S5 and S6, the reaction time of nanoceria with the subDNA and nanoceria with the TMB substrate were 30 and 20 min, respectively. We also explored various commonly used biological buffers to compare their effects on the inhibition of nanoceria’s peroxidase-mimicking activity. Notably, our buffer condition exhibited the highest peroxidase-mimicking activity of bare nanoceria and strongly retained the signal from binding subDNA to the nanoceria (Figure S7). In contrast, in the phosphate buffer, it was confirmed that the peroxidase-mimicking activity of nanoceria was significantly suppressed, regardless of the presence of the subDNA, due to the strong adsorption of phosphate on the nanoceria surface [36].

### 2.4. Evaluation of the Exo III Detection System Performance

**Figure 5:**
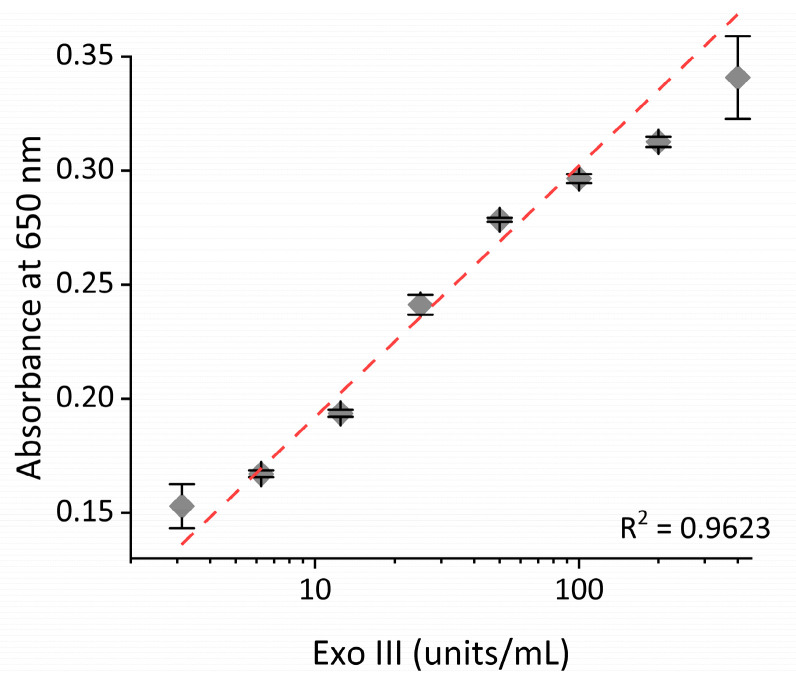
Sensitivity of the nanoceria-based Exo III activity assay. The relationship between the absorbance intensity at 650 nm and the concentration of Exo III. The error bars indicate the standard deviations obtained from triplicate measurements.

**Figure 6:**
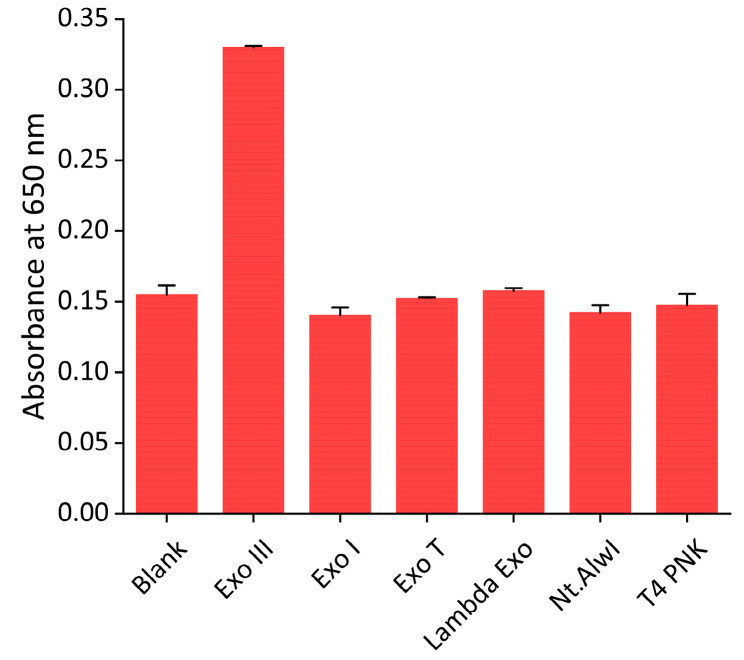
Selectivity of the nanoceria-based Exo III activity assay. The absorbance intensities at 650 nm were measured in the sample containing Exo III (400 units/mL) and other nucleases, such as Exo I, Exo T, Lambda Exo, Nt AlwI, and T4 PNK (400 units/mL). The error bars indicate the standard deviations obtained from triplicate measurements.

To determine the sensitivity of the proposed system, Exo III, under varying concentrations, was subjected to the assay. As shown in Figure 5, the absorbance intensity increased as the concentration of Exo III increased. In addition, a strong linear relationship (R^2^ = 0.9623) was established between the absorbance intensity and the logarithm of the concentration of Exo III from 3.1 to 400 units/mL, respectively. This finding confirms that our technique can quantitively and reliably determine Exo III levels over a wide dynamic range. Based on the 3σ/slope (where σ is the standard deviation of a 3.1 units/mL Exo III), the limit of detection (LOD) was estimated to be 0.263 units/mL. The sensitivity of this approach is comparable to previously reported Exo III detection techniques but exhibits a uniquely wide dynamic range (as detailed in Table 1). Next, the selectivity of our technique was assessed through examining the extent of the absorbance intensity by other nucleases, such as Exo I, Exo T, Lambda Exo, Nt. AlwI, and T4 PNK under the same conditions. As exhibited in Figure 6 and Figure S8, a high absorbance intensity was only observed from Exo III, whereas other nucleases produced only minor absorbance intensities similar to that of the blank. These obtained results clearly demonstrate the remarkable specificity of the proposed strategy towards the intended target, Exo III, thereby validating the high specificity of this system.

### 2.5. Real-Sample Test of the Exo III Detection System

To verify the general applicability of this system for biological samples, such as human serum, Exo III under varying concentrations was spiked into 10% human serum and subjected to the proposed assay. As shown in Figure S9, an excellent linear relationship (R^2^ = 0.9852) was also obtained between the absorbance intensity and the logarithm of the concentration of Exo III from 6.2 to 400 units/mL, respectively, in 10% human serum. Based on this calibration curve, the concentrations of Exo III in 10% human serum were successfully determined, with a coefficient of variation (CV) of less than 3.382% and a recovery ratio between 98.74 and 107.53%, respectively (Table 2). Overall, these results demonstrate that this developed Exo III detection system is able to identify Exo III in complex heterogeneous specimens, thereby verifying its robust applicability for real biological sample analysis.

## 3. Materials and Methods

### 3.1. Materials

The oligonucleotide sequences used in this work (Table S1) and nuclease-free water were purchased from Integrated DNA Technologies (Coralville, IA, USA). Moreover, 10× NEBuffer 1, exonuclease I, exonuclease III, exonuclease T, lambda exonuclease, Nt. AlwI, and T4 polynucleotide kinase were all purchased from New England Biolabs (Ipswich, MA, USA). Nanoceria dispersion (catalog number 289744, 20 wt% dispersed in 2.5% acetic acid), 3,3′,5,5′-tetramethylbenzidine (TMB, catalog number T0440), human serum (catalog number H4522), 1 M HEPES buffer solution (pH 5.0) (Figure S7), sodium phosphate dibasic (Figure S7), sodium phosphate monobasic (Figure S7), acetic acid (Figure S7), and sodium acetate (Figure S7) were all purchased from Sigma-Aldrich (St. Louis, MO, USA). Finally, 1 M Tris-HCl buffer (pH 7.4) (Figure S7) was purchased from Bioneer (Daejeon, Republic of Korea).

### 3.2. Nanoceria-Based Exonuclease III Assay

The solution was prepared by mixing 2.5 μL of NEBuffer 1 (10×), 7.5 μL of nuclease-free water, 10 μL of substrate DNA (1 μM), and 5 μL of the sample containing the exonuclease. The mixture was then incubated at 37 °C for 1 h. After incubation, 20 μL of the reaction mixture was mixed with 60 μL of nanoceria (0.5 wt%) and incubated at room temperature for 30 min to permit the aggregation of the substrate DNA and nanoceria. Then, 100 μL of TMB was added and incubated at room temperature for 20 min followed by centrifugation at 10,000× *g* for 1 min to separate the nanoceria from the solution. The absorbance intensity was measured using 100 μL of supernatant. Spectrophotometric measurements were carried out using the Infinite M 200 PRO (Tecan, Zurich, Switzerland) equipped with a desktop computer for absorbance data acquisition.

### 3.3. Confirmation of DNA-Induced Aggregation of Nanoceria

The scanning electron microscopy images were obtained using an emission scanning electron microscope (FE-SEM, Hitachi SU-8010, Hitachi, Japan) at a voltage of 5 kV. Particle size distribution and zeta potential measurements were performed using a Zetasizer Lab (Malvern Panalytical Ltd., Malvern, UK), and the samples were scanned three times to obtain the average zeta potential and diameter of the nanoceria.

### 3.4. Gel Electrophoresis Analysis of the Nuclease Reactant

The solution was prepared by mixing 2.5 μL of NEBuffer 1 (10×), 7.5 μL of nuclease-free water, 10 μL of substrate DNA (1 μM), and 5 μL of the sample containing various nucleases (Exo III, Exo I, Exo T, Lambda Exo, Nt. AlwI, and T4 PNK). The mixture was then incubated at 37 °C for 1 h. A mixture of 5 μL of nuclease reactant and 1 μL of gel electrophoresis loading buffer (6×, Bioneer) was loaded onto a 2% agarose gel, and gel electrophoresis was conducted at 100 V for 50 min using 1× TBE as the running buffer. After staining with GreenStar™ nucleic acid staining solution I (1×, Bioneer), a gel image was photographed using a Gel Doc Go Imaging System (Bio-Rad, Hercules, CA, USA).

### 3.5. Real-Sample Test of the Exonuclease III Detection System

Various concentrations of Exo III were spiked into 10% human serum to mimic real samples. To quantify the amount of spiked Exo III, a calibration curve was constructed using a standard set of known concentrations of Exo III in the 10% human serum. The unknown quantity of Exo III was then estimated based on this curve.

## 4. Conclusions

In this study, we developed a label-free colorimetric assay to detect levels of Exo III activity based on the peroxidase-mimicking activity of nanoceria. This strategy was based on the mechanism by which the peroxidase-mimicking activity of nanoceria was inhibited by binding with the subDNA. The presence of Exo III causes subDNA degradation and promotes the peroxidase-mimicking activity of nanoceria, increasing the absorbance intensity. Based on this strategy, Exo III was successfully detected at an LOD of 0.263 units/mL; a wide dynamic range from 3.1 to 400 units/mL, respectively, along with an excellent specificity was also confirmed. In contrast to previously reported studies, this strategy offers a greater level of feasibility as it does not rely on fluorescence dye labels and does not necessitate intricate DNA design nor sophisticated experimental techniques.

## Figures and Tables

**Table 1:** Comparison of the different methods that have been used for the determination of Exo III activity.

Method	Limit Detection (units/mL)	Linear Range (units/mL)	Ref.
Tb^3+^	0.8	5–100	[15]
CuNPs	0.02	0.05–2	[20]
ThT	0.5	0–10	[13]
SYBR Green I	0.7	1–200	[37]
Homo-FRET	0.17	0.25–8	[16]
Luminescent	1	0–25	[12]
Graphene oxide	0.001	0.01–0.5	[19]
Nanoceria	0.263	3.1–400	This work

**Table 2:** Determination of Exo III in 10% human serum samples.

Added Exo III (units/mL)	Measured Exo III (μg/mL) ^a^	SD ^b^	CV (%) ^c^	Recovery (%) ^d^
100	98.74	3.784	3.832	98.74
50	53.76	0.226	0.420	107.53

^a^ Mean of three measurements. ^b^ Standard deviation. ^c^ Coefficient of variation = (standard deviation)/mean × 100. ^d^ Recovery = (measured Exo III)/(added Exo III) × 100.

## Data Availability

Not applicable.

## Supplementary Materials

The supporting information can be downloaded at: https://www.mdpi.com/article/10.3390/ijms241512330/s1.
