# Supplementary material for: Peroxidase-Mimicking Activity of Nanoceria for Label-Free Colorimetric Assay for Exonuclease III Activity
Source: Int J Mol Sci. 2023 Aug 2;24(15):12330. doi: 10.3390/ijms241512330 (PMC10418927; doi:10.3390/ijms241512330)
Supplement: Supplementary file 1 [file ijms-24-12330-s001.zip › ijms-2493271-supplementary.pdf]

International Journal of *Molecular Sciences*

MDPI

*Article*

# Peroxidase-mimicking activity of nanoceria for label-free colorimetric assay for exonuclease III activity

**Hyogu Han [1], Jae Hoon Jeung [1,2], Se Hee Jang [1,3], Chang Yeol Lee [4,*], Jun Ki Ahn [1,*]**

[1] Material & Component Convergence R&D Department, Korea Institute of Industrial Technology (KITECH), Ansan 15588, Republic of Korea
[2] Department of Chemistry, Gangneung-Wonju National University, Gangneung 25457, Republic of Korea
[3] Department of Medical Device Engineering and Management, College of Medicine, Yonsei University, Seoul 03722, Republic of Korea
[4] Bionanotechnology Research Center, Korea Research Institute of Bioscience and Biotechnology (KRIBB), Daejeon 34141, Republic of Korea
* Correspondence: lcyeol8457a@kribb.re.kr (C.Y.L); jkahn@kitech.re.kr (J.K.A)

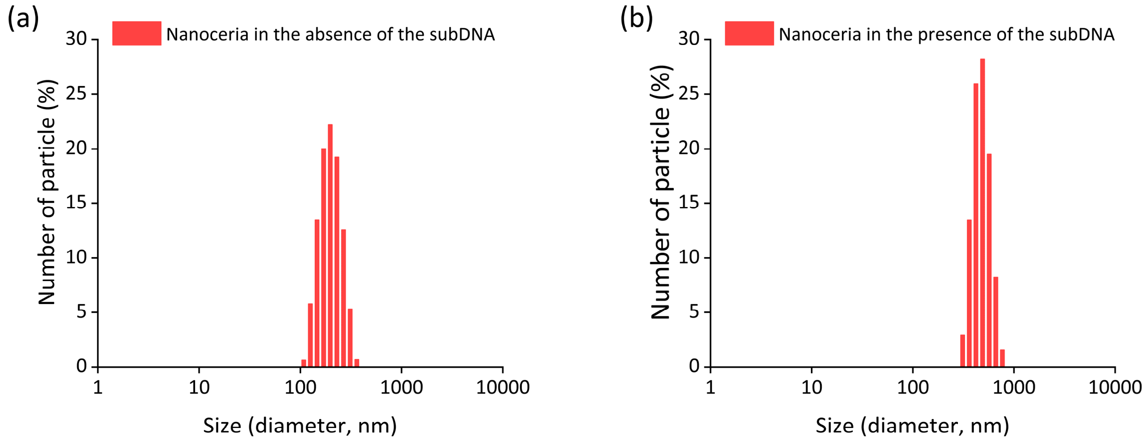


| | Nanoceria in the absence of the subDNA | Nanoceria in the presence of the subDNA |
|---|---|---|
| **Mean diameter (nm)** | 202 | 482 |
| **Zeta potential (mV)** | 7.03 | -1.078 |

**Figure S1.** Characterization of nanoceria in the presence and absence of subDNA. a) particle size distribution analysis by measuring dynamic light scattering of nanoceria in absence and presence of the subDNA. b) mean diameter (nm) and zeta potential (mV) measurements.

**Citation:** To be added by editorial staff during production.

Academic Editor: Firstname Lastname

Received: date
Revised: date
Accepted: date
Published: date

**Copyright:** © 2023 by the authors. Submitted for possible open access publication under the terms and conditions of the Creative Commons Attribution (CC BY) license (https://creativecommons.org/licenses/by/4.0/).

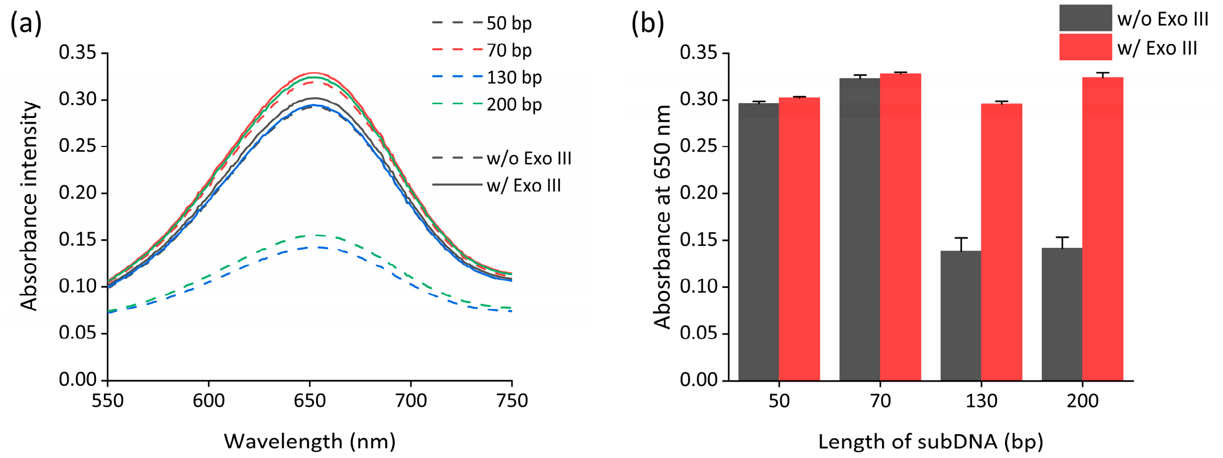


**Figure S2.** Optimization of subDNA length. (a) absorption spectra. (b) absorbance intensity at 650 nm. The error bars indicated the standard deviations obtained from triplicate measurements.

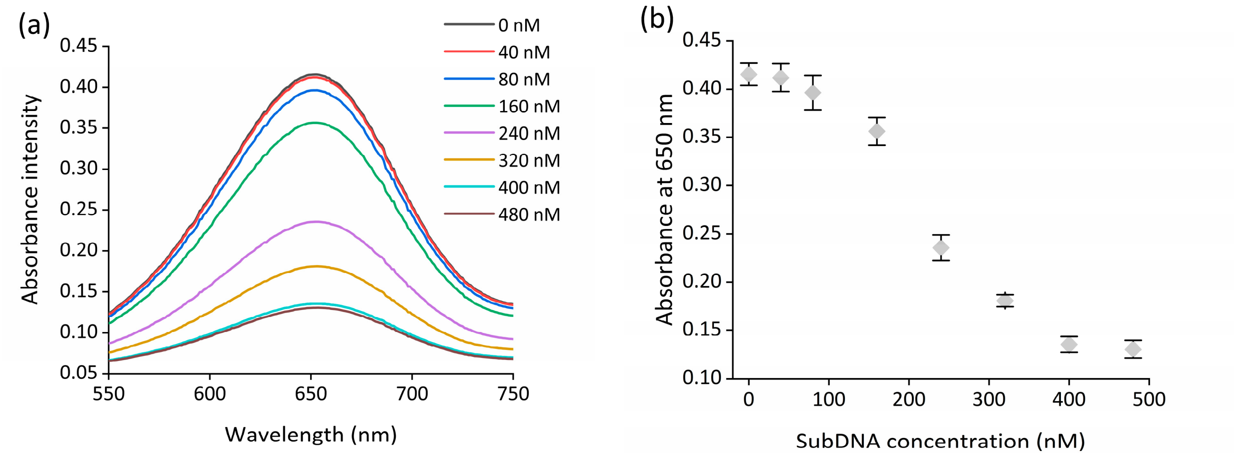


**Figure S3.** Optimization of subDNA concentration. (a) absorption spectra. (b) absorbance intensity at 650 nm. The error bars indicated the standard deviations obtained from triplicate measurements.

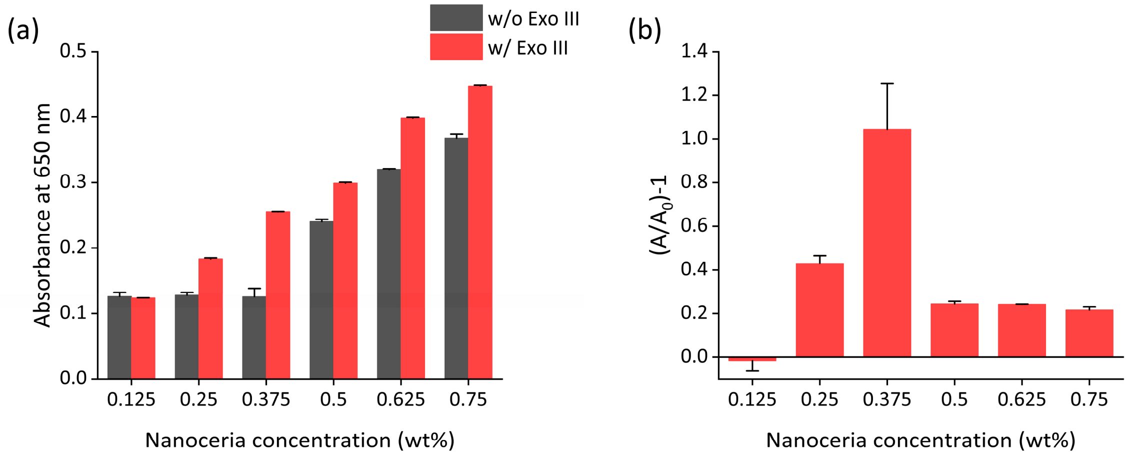


**Figure S4.** Optimization of nanoceria concentration. (a) absorbance intensity at 650 nm. (b) absorbance intensity ratio. The absorbance intensity ratio is defined as $(A/A_0)$-1, where A and $A_0$ indicate the absorbance intensity at 650 nm with and without the Exo III, respectively. The error bars indicated the standard deviations obtained from triplicate measurements.

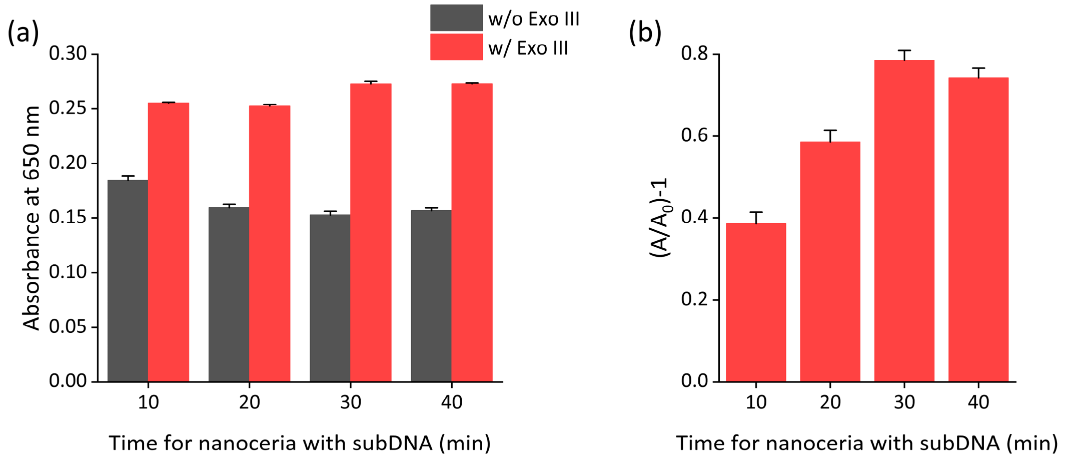


**Figure S5.** Optimization of reaction time of nanoceria with subDNA. (a) absorbance intensity at 650 nm. (b) absorbance intensity ratio. The absorbance intensity ratio is defined as $(A/A_0)$-1, where A and $A_0$ indicate the absorbance intensity at 650 nm with and without the Exo III, respectively. The error bars indicated the standard deviations obtained from triplicate measurements.

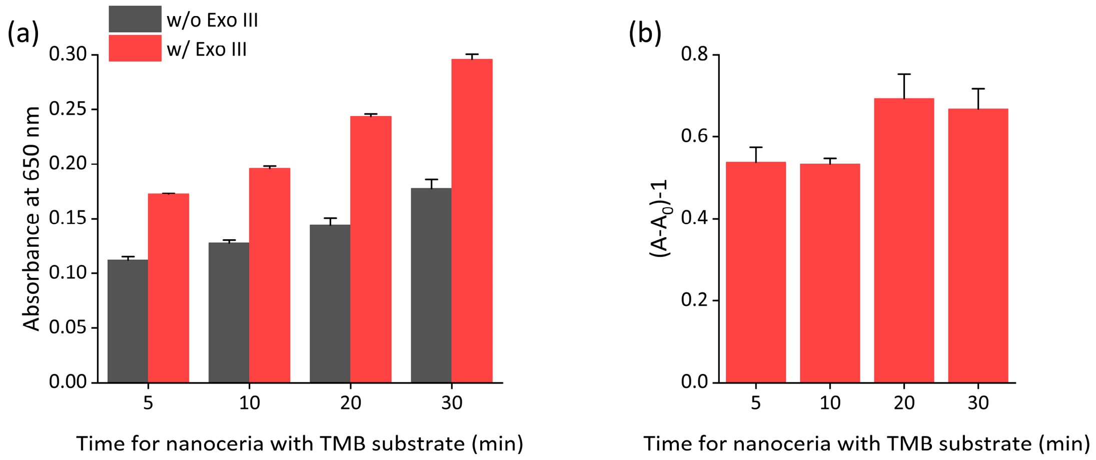


**Figure S6.** Optimization of reaction time of nanoceria with TMB substrate. (a) absorbance intensity at 650 nm. (b) absorbance intensity ratio. The absorbance intensity ratio is defined as $(A/A_0)-1$, where A and $A_0$ indicate the absorbance intensity at 650 nm with and without the Exo III, respectively. The error bars indicated the standard deviations obtained from triplicate measurements.

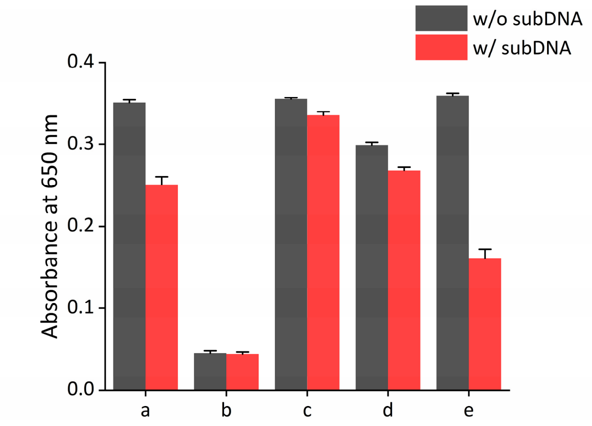


**Figure S7.** Peroxidase-mimicking activity inhibition of subDNA at various buffers. a: 100 mM HEPES buffer pH 5.5, b: 100 mM phosphate buffer pH 7.4, c: Tris-HCl buffer pH 7.4, d: 400 mM acetate buffer pH 4.0, e: 70 mM acetate buffer pH 4.0 (this work). The nanoceria, subDNA concentrations were 0.375 wt%, 400 nM. The error bars indicate the standard deviations obtained from triplicate.

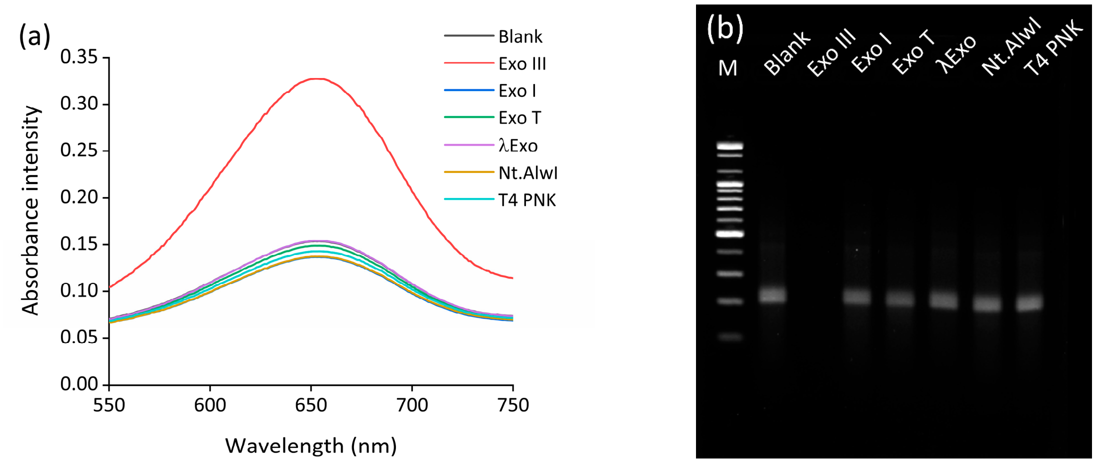


**Figure S8.** Selectivity of Selectivity of the nanoceria based Exo III activity assay. (a) absorption spectra. (b) agarose gel electrophoresis.

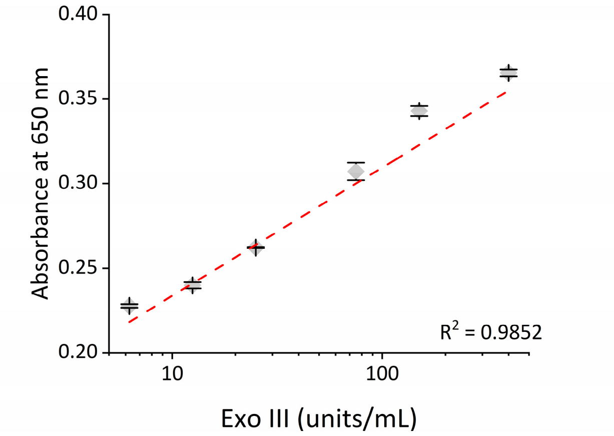


**Figure S9.** The absorbance intensity as a function of Exo III concentrations in 10% human serum. The error bars indicate the standard deviations obtained from triplicate.

**Table S1.** Oligonucleotide sequences employed in this study.

| Name | Sequence (5′-3′) |
|---|---|
| subDNA | TCCGACGTTAAGTACATTACCCTGTCATAGGCGGCGTTCAGGATCACGTTACCGCCATATGATGCGAGCATGACATCATCTCCGCTGTGCCCACCCCAGTAGTGATTATTCCTATAACCCTTCTGAGTGTCCGGAGGCGGAAATCCGCCACGAATGAGAATGTATTTCCCCGACAATCATAATGGGGCGCTCCTAAGCGG |
| subDNA complementry | CCGCTTAGGAGCGCCCCATTATGATTGTCGGGGAAATACATTCTCATTCGTGGCGGATTTCCGCCTCCGGACACTCAGAAGGGTTATAGGAATAATCACTACTGGGGTGGGCACAGCGGAGATGATGTCATGCTCGCATCATATGGCGGTAACGTGATCCTGAACGCCGCCTATGACAGGGTAATGTACTTAACGTCGGA |
